# Nasal and Pharyngeal Colonization by Bacterial Pathogens: A Comparative Study between Preclinical and Clinical Sciences Medical Students

**DOI:** 10.1155/2018/7258672

**Published:** 2018-06-06

**Authors:** Dharm Raj Bhatta, Deependra Hamal, Rajani Shrestha, Ranjana Parajuli, Nisha Baral, Supram Hosuru Subramanya, Niranjan Nayak, Shishir Gokhale

**Affiliations:** Department of Microbiology, Manipal College of Medical Sciences, Pokhara, Nepal

## Abstract

**Background:**

Upper respiratory tract is one of the commonest sites for microbial colonization. The colonized individuals are at risk of infections and can be a source of transmission of pathogens. Medical students are frequently exposed to a variety of infectious agents and more likely to get colonized by them. This study was aimed to determine the prevalence and to compare the colonization rates of nasal and pharyngeal bacterial pathogens among preclinical and clinical sciences medical students.

**Methods:**

This cross-sectional study was conducted among 100 preclinical and 100 clinical sciences medical students. Isolation, identification, and antibiotic susceptibility testing of the isolates were performed by standard microbiological techniques.

**Results:**

The nasal colonization by *S. aureus* and MRSA was 35% (70/200) and 19.5% (39/200), respectively. The nasal colonization by *S. aureus* and MRSA was significantly higher among clinical sciences students as compared to preclinical sciences students. Pharyngeal colonization by *Haemophilus influenzae* was significantly higher among clinical sciences students as compared to preclinical sciences students. The pharyngeal colonization by beta-hemolytic streptococci (nongroup A) was higher among preclinical sciences students than clinical sciences students.

**Conclusion:**

The nasal colonization by *S. aureus* and MRSA was higher among clinical sciences students. Pharyngeal colonization by potential bacterial pathogens was higher among clinical sciences students than preclinical students. Periodic screening of MRSA and potential throat pathogens of clinical sciences students and may reduce the incidences of nosocomial transmission of pathogens.

## 1. Introduction

Respiratory tract infections are one of the important causes of morbidity and mortality worldwide [1]. Upper respiratory tract, perpetually exposed to the environmental flora, is prone to get colonized with a variety of microbial agents. Bacterial pathogens such as *Streptococcus pneumoniae*, *Streptococcus pyogenes*, *Haemophilus influenzae*, *Neisseria meningitidis*, and *Staphylococcus aureus* often colonize the human upper respiratory tract. These pathogens get easily transmitted from colonized/infected individuals to healthy population via respiratory droplets or oropharyngeal secretions. Asymptomatic colonization by potential pathogens like *Streptococcus pneumoniae*, *Streptococcus pyogenes*, *Haemophilus influenzae*, and *Neisseria meningitidis* may lead to invasive infections and serious complications. The carrier rates of these pathogens vary depending upon socioeconomic status, environmental conditions, age, and antimicrobial therapy of the population [2]. Increasing resistance to antimicrobial agents and emergence of multidrug resistant (MDR) organisms makes the treatment of invasive infections challenging [3].

Transmission of respiratory pathogens in healthcare setting is determined by interaction with patients and microbial load in the hospital environment. Environmental quality of healthcare setting and concentration of airborne microbes depend on microbes expelled by patients and healthcare workers. In absence of adequate ventilation, microbes may persist in the hospital environment for variable durations. This enhances the risk for occupants, patients, and medical students who spend long hours in the hospital environment. The individuals involved in patient care and medical students of clinical sciences have greater chances of carrying pathogenic bacteria and can be a potential source of nosocomial infections [4–7].

This study was planned to determine the nasal and pharyngeal colonization by bacterial pathogens among undergraduate medical students and to compare the difference in the colonization rates among preclinical and clinical sciences medical students. We hypothesized that exposure to hospital environment would increase nasal and pharyngeal bacterial colonization among students of clinical sciences as compared to preclinical sciences students who have no such exposure to hospital environment. Similarly, higher antibiotic resistance pattern among isolates from clinical sciences students as compared to preclinical students is expected.

## 2. Materials and Methods

### 2.1. Study Design and Subjects

This cross-sectional prospective study was conducted over a period of five months from May 2017 to September 2017. Approval from the Institutional Ethical Committee (IRC) of Manipal College of Medical Sciences (MCOMS), Pokhara, Nepal, was obtained before the commencement of the study. All participants were enrolled after obtaining the written informed consent. Study population comprised 200 undergraduate medical students. The total available study population in one batch of preclinical sciences (Subset A) was 100 students. Therefore, whole population of one batch was included. Equal number of students from clinical sciences (Subset B) was taken for a comparative study after consulting a statistician. Basic and clinical sciences blocks are separated by a distance of 3.5 km. The students from both subsets do not share residential or mess arrangements and have very infrequent interactions.

#### 2.1.1. Inclusion Criteria

Clinical science students with minimum two years of hospital exposure (interns) were included in Subset B. Preclinical sciences students without histories of hospitalization in the last six months were included in Subset A.

#### 2.1.2. Exclusion Criteria

Clinical sciences students who were on long leave (>2 months) and preclinical sciences students with a history of hospitalization within the last six months were excluded from the study.

Demographic details including age, gender, nationality, hospital exposure, recent exposure to antibiotics, respiratory illness, smoking, chewing tobacco/gutka, and so on were collected by a structured questionnaire. The data of the related isolates from the Clinical Microbiology laboratory of Manipal Teaching Hospital were collected to determine the prevalence of respiratory pathogens among the patients for analysis during the study period.

### 2.2. Specimen Collection

One specimen from the anterior nasal cavity and one from the posterior pharyngeal wall (200 nasal swabs and 200 pharyngeal swabs) from 200 participants (100 from Subset A and 100 from Subset B) were collected. Specimens were obtained by rubbing both the anterior nares with the first swab and by swabbing the posterior oropharynx by the second swab.

### 2.3. Isolation and Identification of Bacterial Isolates

Specimens were inoculated immediately on 5% sheep blood agar and chocolate agar (Hi Media, Mumbai, India) plates at the point of collection. Inoculated plates were immediately transported to the laboratory and incubated at 37°C in a candle jar with increased CO_2_ partial pressure. Identification of the isolates was performed by standard microbiological techniques such as colony morphology, Gram stain, the catalase test, the coagulase test, optochin sensitivity, bacitracin sensitivity, and the satelitism test [8].

### 2.4. Antibiotic Susceptibility Test

Antibiotic susceptibility testing of the isolates was performed on Mueller–Hinton agar (HI Media, Mumbai, India) by the Kirby–Bauer disc diffusion method [9]. Methicillin resistant *Staphylococcus aureus* (MRSA) was screened by the cefoxitin (30 *µ*g) disc diffusion method [9].

### 2.5. Statistical Analysis

The correlation between variables was determined by the chi-square test using SPSS 11.5. A *p* value ≤ 0.05 was considered significant.

## 3. Results

A total of 200 medical (MBBS) students comprising 100 students in Subset A (first year) and 100 in Subset B (interns) were enrolled in this study. The Subset A participants were 18–25 years of age, while Subset B were between 22 and 30 years of age. Demographic details of the students are shown in Table 1.

Bacteriological profile of nasal and pharyngeal swab cultures of Subset A and Subset B is shown in Table 2. The overall nasal colonization by *S. aureus* and MRSA was 35% (70/200) and 19.5% (39/200), respectively. The nasal colonization rate of *S. aureus* and MRSA was significantly higher (*p* values 0.003 and 0.001) among Subset B as compared to Subset A (Table 2). The isolates from the pharynx (Subset A and Subset B) were *Haemophilus influenzae* (12.5%), nongroup A beta-hemolytic streptococci (5.5%), *S. aureus* (5.5%), *Streptococcus pneumoniae* (4%), and *Haemophilus parainfluenzae* (3.5%). Pharyngeal colonization by *Haemophilus influenzae* was found significantly higher (*p* value 0.005) among Subset B as compared to Subset A (Table 2). Among the participants, 7.5% (15/200) were colonized with *S. aureus* in the nasal cavity along with other potential pathogens in the pharynx (*Haemophilus influenzae*, beta-hemolytic Streptococci, and *Streptococcus pneumoniae*).

Out of 100 nasal swabs collected from Subset A, *S. aureus* was isolated from 25 (25%) participants, of which 10 (40%) were MRSA. The nasal carriage rate of *S. aureus* was 28.8% (17/59) and 19.5% (8/41) among male and female participants, respectively, which was statistically insignificant (*p* value 0.291). Similarly, the nasal carriage rate of MRSA was 13.5% (8/59) and 4.9% (2/41) among male and female participants, respectively. This difference was statistically insignificant (*p* value 0.155). Other bacterial flora isolated from nasal swabs includes *Staphylococcus epidermidis*, *Micrococcus* species, *Enterococcus* species, and aerobic spore bearer.

Bacteriological culture of nasal and pharyngeal specimens among Subset B showed similar bacterial isolates as in Subset A but relatively higher rate of colonization (Table 2). Nasal colonization by *S. aureus* among Subset B was 45% (45/100) with 29% (29/100) MRSA. The nasal carriage rate of *S. aureus* among male and female was 41% (25/61) and 41% (16/39), respectively. Similarly, the nasal MRSA colonization rate among participants was 26.2% (16/61) in male and 33.3% (13/39) in females. Antibiotic resistance pattern of *S. aureus* isolates from Subset A and Subset B is depicted in Table 3. No significant difference in the antibiotic resistance pattern of *S. aureus* isolates from Subset A and Subset B was observed. Antibiotic resistance pattern of pharyngeal isolates is depicted in Table 4.

Majority of students with nasal carriage of *S. aureus* had no history of antibiotic use in the past two weeks (95.8%), no history of hospitalization in the past six months (98.6%), no history of respiratory infections (92.9%), and no history of smoking/tobacco intake (95.7%). Similarly, majority of students with pharyngeal carriage of potential bacterial pathogens had no history of antibiotic use, hospitalization, respiratory infection, and smoking/tobacco intake.

## 4. Discussion

Burden of nosocomial infections is significantly higher in developing countries [10]. Healthcare professionals are considered important reservoirs for transmission of nosocomial pathogens. Another potential source of infections could be medical students particularly those involved in patient care. Human nasal cavity and pharynx are favorable anatomical sites for colonization by microbial agents. Therefore, it is important to screen healthcare workers and medical students for colonization by bacterial pathogens.

In our study, the nasal carriage rate of *S. aureus* among the clinical science student (Subset B) was 45% which is significantly higher than the preclinical science (Subset A) student (25%). Findings of our study are comparable with other studies with the colonization rate around 40% [11, 12]. In a similar study conducted among preclinical sciences students of Nepal, 15% of nasal carriage of *S. aureus* was reported [13]. However, similar studies from other countries have shown colonization rates ranging from 19% to 25% [14–16]. The nasal carriage rate of MRSA among Subset B in our study was 29% which is significantly higher than Subset A (10%). The MRSA colonization rate reported in 2016 by Ansari et al. from Nepal was lower (4%) than our findings [13]. Lower MRSA colonization rates ranging from 0% to 9.4% have been reported from other countries [14, 15, 17, 18]. Higher rates of *S. aureus* colonization among medical students of clinical sciences can be attributed to long-term exposure to hospital environment and regular interaction with patients. However, Syafina et al. reported higher *S. aureus* colonization among preclinical students as compared to clinical students [19]. Pathogens like MRSA survive for long durations due to their stability in the hospital environment. This enhances the risk of transmission and colonization. The MRSA colonization rates among healthcare workers in a particular setting depend on local prevalence of MRSA and infection control practices. High percentage of MRSA colonization among clinical students in our study could be associated with the high prevalence of MRSA (34.75%) among the clinical isolates of the Manipal Teaching hospital [20]. High prevalence of MRSA among the patients in healthcare setting increases the risk of colonization among the healthcare workers due to frequent interactions. Preclinical students on the other hand are away from the hospital environmental exposure and have lower *S. aureus* and MRSA colonization.

The carriage rate of *S. aureus* and MRSA among male students was found higher as compared to females which are comparable with other reports [15, 21, 22]. Older age has been considered as a possible factor for *S. aureus* colonization. The clinical science student of older age group is colonized more with *S. aureus* as compared to the younger age preclinical student.

Antibiograms of *S. aureus* isolated from Subset B showed higher but statistically insignificant resistance pattern as compared to Subset A (Table 2). This could be correlated with the fact that hospital strains are more resistant to commonly used antibiotics as compared to community strains [23, 24]. The antibiotic resistance pattern of the isolates is likely to change over a period of time due to changes in antibiotic prescription pattern. Increased antimicrobial resistance has been reported from the isolates of tertiary care teaching hospitals, possibly due to increased selective pressure arising from widespread antimicrobial use, high density of patients and healthcare workers, inadequate ventilation, and inadequate infection control policies. *S. aureus* isolates in our study were found more resistant to commonly used antibiotics as compared to report by Okamo et al. [15].

Upper respiratory tract colonization by bacterial pathogens increases the risk of invasive infections and transmission to the susceptible host. Microbial colonization is a complex process which involves colonization, elimination, and recolonization [25]. The overall pharyngeal colonization rate by bacterial pathogens was higher among Subset B as compared to Subset A but was statistically not significant except in case of *H. influenza*e. Higher rates of pharyngeal colonization among Subset B could be due to prolonged (>2 years) hospital environmental exposure and interactions with the variety of patients. The pharyngeal colonization rate of beta-hemolytic streptococci was higher among Subset A but statistically insignificant. Previous study from the same institution reported 65% colonization rate with *Streptococcus pneumoniae* and/or *Haemophilus* species among the healthcare workers which is higher than our findings [26]. Number of contributing factors could be associated with the nasal and pharyngeal microbial colonization. The common risk factors include younger age, winter, overcrowding, low socioeconomic status, and smoking. Low pharyngeal colonization rate in our study may be due to the fact that majority of the participants were nonsmokers belonging to middle and high income groups and specimens were collected in summer. The data from Clinical laboratory of Manipal Teaching Hospital, for corresponding months, revealed low rate of isolation of abovementioned organisms from the clinical samples. Lower prevalence of these pathogens among the patients could be related to lower pharyngeal carriage rates among the students. The nasal colonization rate of *S. aureus* was higher as compared to potential pathogens isolated from the pharynx. Pathogens like *Streptococcus pneumoniae*, *Streptococcus pyogenes*, and *Haemophilus influenzae* are delicate and get inactivated shortly after excretion and exposure to disinfectants. The survival time of these organisms in the hospital environment is shorter as compared to MRSA reflecting their poor transmission and low colonization rates.

The overall antibiotic resistance pattern of the pharyngeal isolates (Subset A and Subset B) revealed that most of the *Haemophilus* isolates were susceptible to commonly used antibiotics. Among *Streptococcus pneumoniae* isolates, 37.5% (3/8) were resistant to penicillin indicating the increasing resistance. Out of 11 beta-hemolytic streptococci, 45.4% (4/11) showed intermediate susceptibility to penicillin. This finding may be alarming for future emergence of penicillin-resistant strains. Finding of our study is comparable with study from India [27].

Screening of nasal and pharyngeal colonization among healthcare workers and medical students may help to predict the transmission of potential pathogens. The risk of transmission and dissemination of bacterial pathogens from one unit/ward to another is more with clinical sciences students as they undergo periodic rotational training program in various wards/units. The clinicians and other healthcare workers work in one particular unit and are less likely to spread the pathogens from one unit to another. Screening data can be important to minimize the transmission among high-risk patients like neonates, burn patients, postoperative patients, and ICU patients. In view of the above, survey of the hospital regarding the prevalence of MRSA and other potential pathogens in different units will help to tackle the situation. Based on the available data, hospital units can be classified as high-risk units (zone), moderate risk-units, and low-risk units. Awareness/orientation program may be needed after completion of the preclinical science study regarding the standard practices to minimize transmission and prevention of hospital-acquired infections. Regular screening program and restriction of colonized healthcare workers and medical students especially in high-risk units should be strictly implemented in order to reduce transmission of nosocomial pathogens.

The strength of our study lies in coverage of nasal and pharyngeal colonization by bacterial pathogens among medical students as most of the previous studies had limited findings of nasal colonization by *S. aureus*/MRSA only. Nasal and pharyngeal colonization among medical students could be a potential source of clonal spread of MRSA in the hospital. This has been amply emphasized in our study.

This study had some limitations. The study was conducted during the autumn/summer season; hence, seasonal variation in the colonization rate could not be studied. Molecular analysis of the potential pathogens was not done. The transmission of pathogens from medical students to patients, healthcare workers, and vice versa was not included in this study. The study was conducted among students of one medical college of Western Nepal, and results of the study cannot be generalized to students of other medical colleges.

## 5. Conclusion

Medical students of clinical sciences have higher rates of *S. aureus* and MRSA colonization. Higher colonization is perhaps due to prolonged hospital environmental exposure and interaction with patients. Pharyngeal colonization by potential pathogens was higher among the clinical sciences student. Higher rates of nasal and pharyngeal colonization among medical students can be threat for transmission of potential pathogens among patients and healthcare workers. Awareness among medical students about their role in transmission of pathogens and importance of universal precautions and hand hygiene would help in reducing the nosocomial infections.

## Figures and Tables

**Table 1:** Demographic and clinical details of the students.

Variable	Subset A (*N*=100) frequency	Subset B (*N*=100) frequency
*Sex*		
Male	59	61
Female	41	39
*Nationality*		
Nepali	62	58
Others (Indians, Sri Lankan, and Maldivian)	38	42
*Antibiotic intake*		
Yes	05	04
No	95	96
*Hospitalization*		
Yes	00	03
No	100	97
*Respiratory infection*		
Yes	06	05
No	94	95
*Smoking/tobacco intake*		
Yes	03	04
No	97	96

**Table 2:** Distribution of bacterial isolates from nasal and pharyngeal specimens.

Organism	Nasal swab Subset A (*N*=100)	Nasal swab Subset B (*N*=100)	*p* value
*Staphylococcus aureus*	25	45	0.003^*∗*^
Methicillin-resistant *Staphylococcus aureus* (MRSA)	10	29	0.001^*∗*^

	Pharyngeal swab Subset A (*N*=100)	Pharyngeal swab Subset B (*N*=100)	

*Staphylococcus aureus*	5	6	0.756
Methicillin-resistant *Staphylococcus aureus* (MRSA)	2	3	0.651
*Haemophilus influenzae*	6	19	0.005^*∗*^
*Haemophilus parainfluenzae*	3	4	0.700
*Streptococcus pneumoniae*	2	6	0.149
Beta-hemolytic streptococci	8	3	0.121

^*∗*^Significant association.

**Table 3:** Antibiotic resistance pattern of *Staphylococcus aureus* isolates from nasal and pharyngeal specimens.

Antibiotic	Subset A (*N*=30) frequency (percentage)	Subset B (*N*=51) frequency (percentage)	*p* value
Penicillin	27 (90%)	50 (98%)	0.107
Erythromycin	16 (53.3%)	38 (74.5%)	0.051
Ciprofloxacin	10 (33.3%)	21 (41.1%)	0.483
Gentamicin	01 (3.3%)	03 (5.8%)	0.609
Clindamycin	06 (20%)	11 (21.5%)	0.867
Ceftriaxone	06 (20%)	14 (27.4%)	0.453
Amikacin	01 (3.3%)	03 (5.8%)	0.609
Tetracycline	02 (6.6%)	06 (11.7%)	0.458
Vancomycin	00	00	—

**Table 4:** Antibiotic resistance pattern of pharyngeal isolates.

Organism (number)	Antibiotics (resistant percentage)						
	P	CIP	COT	C	CTR	AZM	E
*Haemophilus influenzae* (25)	—	1 (4%)	3 (12%)	1 (4%)	00	5 (20%)	—
*Haemophilus parainfluenzae* (7)	—	1 (14.3%)	1 (14.3%)	00	00	00	—
Beta-hemolytic streptococci (11)	00^*∗*^	00	00	—	00	—	—
*Streptococcus pneumoniae* (8)	3 (37.5%)	00	00	—	00	—	2 (25%)

^*∗*^Four isolates of beta-hemolytic streptococci (45.4%) were intermediate sensitive to penicillin. AZM: azithromycin, CIP: ciprofloxacin, CTR: ceftriaxone, COT: cotrimoxazole, C: chloramphenicol, E: erythromycin, P: penicillin.

## Data Availability

The data used to support the findings of this study are available from the corresponding author upon request.

## References

[1] GBD 2013 Mortality and Causes of Death Collaborators (2015). Global, regional, and national age–sex specific all-cause and cause-specific mortality for 240 causes of death, 1990–2013: a systematic analysis for the global burden of disease study 2013. *The Lancet*.

[2] Masuda K., Masuda R., Nishi J. N., Tokuda K., Yoshinaga M., Miyata K. (2002). Incidence of nasopharyngeal carriage of respiratory bacterial pathogens in Japanese children attending day care centers. *Pediatrics International*.

[3] Akova M. (2016). Epidemiology of antimicrobial resistance in blood stream infections. *Virulence*.

[4] Guclu E., Yavuz T., Tokmak A. (2007). Nasal carriage of pathogenic bacteria in medical students: effects of clinic exposure on prevalence and antibiotic susceptibility. *European Archives of Oto-Rhino-Laryngology*.

[5] Eveillard M., Martin Y., Hidri N., Boussougant Y., Joly-Guillou M. L. (2004). Carriage of methicillin-resistant *Staphylococcus aureus* among hospital employees: prevalence, duration, and transmission to households. *Infection Control and Hospital Epidemiology*.

[6] Adesida S. A., Abioye O. A., Bamiro B. S. (2007). Associated risk factors and pulsed field gel electrophoresis of nasal isolates of *Staphylococcus aureus f*rom medical students in a tertiary hospital in Lagos, Nigeria. *Brazilian Journal of Infectious Diseases*.

[7] Ma X. X., Sun D. D., Wang S. (2011). Nasal carriage of methicillin-resistant *Staphylococcus aureus* among preclinical medical students: epidemiologic and molecular characteristics of methicillin-resistant *S. aureus* clones. *Diagnostic Microbiology and Infectious Disease*.

[8] Cheesbrough M. (2006). *District Laboratory Practice in Tropical Countries*.

[9] Clinical and Laboratory Standards Institute (2017). *Performance Standards for Antimicrobial Susceptibility Testing, Twenty-Second Informational Supplement*.

[10] Danasekaran R., Mani G., Annadurai K. (2014). Prevention of healthcare-associated infections: protecting patients, saving lives. *International Journal of Community Medicine and Public Health*.

[11] Shaza B. R., Bansal V. P., Bhalchandra M., Mishra J. (2017). *Staphylococcus aureus* nasal carriers and the prevalence of methicillin resistant *Staphylococcus aureus* among medical students. *International Journal of Research in Medical Sciences*.

[12] Lopez-Aguilera S., María G. Y., Barrado L., Salinas M. C. G. R., Otero J. R., Chaves F. (2013). *Staphylococcus aureus* nasal colonization in medical students: importance in nosocomial transmission. *Enfermedades Infecciosas y Microbiología Clínica*.

[13] Ansari S., Gautam R., Shrestha S., Ansari S. R., Subedi S. N., Chhetri M. R. (2016). Risk factors assessment for nasal colonization of *Staphylococcus aureus* and its methicillin resistant strains among pre-clinical medical students of Nepal. *BMC Research Notes*.

[14] Javaeed A., Khan S. H., Haqsubhani R., Ghauri S. K., Jahan S. (2016). Methicillin resistant *Staphylococcus aureus* prevalence of nasal carriage among healthy MBBS students of Continental Medical College, Lahore. *Pakistan Journal of Medical and Health Science*.

[15] Okamo B., Moremi N., Seni J., Mirambo M. M., Kidenya B. R., Mshana S. E. (2016). Prevalence and antimicrobial susceptibility profiles of *Staphylococcus aureus* nasal carriage among pre-clinical and clinical medical students in a Tanzanian University. *BMC Research Notes*.

[16] Bettin A., Causil C., Reyes N. (2012). Molecular identification and antimicrobial susceptibility of *Staphylococcus aureus* nasal isolates from medical students in Cartagena, Colombia. *Brazilian Journal of Infectious Diseases*.

[17] Treesirichod A., Hantagool S., Prommalikit O. (2014). Nasal carriage and antimicrobial susceptibility of *Staphylococcus aureus* among medical students at the HRH Princess Maha Chakri Sirindhorn Medical Center, Thailand: a follow-up study. *Journal of Infection and Public Health*.

[18] Srivastava N., Goyal A., Goyal S., Kumar R., Gupta B. R. (2015). Comparative study on prevalence of nasal carriage of MRSA and MSSA in medical students with clinical posting and without clinical posting: is introduction of hospital infection control policy in medical curriculum need of hour. *IOSR Journal of Dental and Medical Sciences*.

[19] Nordin S. A., Path M., Zaim N. A. N. (2012). *Staphylococcus aureus* nasal carriers among medical students in a medical school. *Medical Journal of Malaysia*.

[20] Bhatta D. R., Cavaco L. M., Nath G., Gaur A., Gokhale S., Bhatta D. R. (2015). Threat of multidrug resistant *Staphylococcus aureus* in Western Nepal. *Asian Pacific Journal of Tropical Disease*.

[21] Chen C. S., Chen C. Y., Huang Y. C. (2012). Nasal carriage rate and molecular epidemiology of methicillin-resistant *Staphylococcus aureus* among medical students at a Taiwanese university. *International Journal of Infectious Diseases*.

[22] Bischoff W. E., Wallis M. L., Tucker K. B., Reboussin B. A., Sherertz R. J. (2004). *Staphylococcus aureus* nasal carriage in a student community: prevalence, clonal relationships, and risk factors. *Infection Control and Hospital Epidemiology*.

[23] Chadha T., Kulsum S. N., Adlekha S., Mailapur P. C. (2014). Comparison of antibiotic susceptibility pattern of community- and hospital-acquired methicillin resistant *Staphylococcus aureus* with special reference to inducible clindamycin resistance in a tertiary care hospital in southern India. *Medical Journal of Dr. D. Y. Patil University*.

[24] Huang H., Flynn N. M., King J. H., Monchaud C., Morita M., Cohen S. H. (2006). Comparisons of community-associated methicillin-resistant *Staphylococcus aureus* (MRSA) and hospital-associated MSRA infections in Sacramento, California. *Journal of Clinical Microbiology*.

[25] Bogaert D., De Grott R., Hermans P. W. (2004). *Streptococcus pneumoniae* colonization: the key to pneumococcal disease. *Lancet Infectious Diseases*.

[26] Subramanya H. S., Thapa S., Dwedi S. K. (2016). *Streptococcus pneumoniae* and *Haemophilus* species colonization in health care workers: the launch of invasive infections?. *BMC Research Notes*.

[27] Dhanda V., Chaudhary P., Toor D., Kumar R., Chakraborty A. (2013). Antimicrobial susceptibility pattern of beta-haemolytic group A, C and G streptococci isolated from North India. *Journal of Medical Microbiology*.

